# Preventing Medicine mistakes in pediatric and neonatal patients

**Published:** 2015

**Authors:** F Izadpanah, H Haddad Kashani, MR Sharif

**Affiliations:** * Food and Drug Laboratory Research Center, Food and Drug Organization, Ministry of Health and Medical Education, Tehran, Iran,; ** Anatomical Sciences Research Center, Kashan University of Medical Sciences, Kashan, Iran,; *** Department of Pediatrics, Kashan University of Medical Sciences, Kashan, Iran

**Keywords:** Medicine mistakes, pediatric and neonatal patients, fatal

## Abstract

Medicine mistakes are significant issues that happen in clinic environments. Several portions make the pediatric community extra sensitive to medicine faults, and possible complexities are rising from medicine board. These involve the various dosage forms of the identical medication availability, inaccurate dosing, absence of regulated dosing regimen, and process development. Electric information like EMBASE, MEDLINE, Global Pharmaceutical Abstracts, ASSIA, PsycINFO, British Nursing Index, CINAHL, were sleeked among 1985 and December 2014. Study choice Inclusion and exclusion standard are used to specify the eligible publications though title investigation followed by abstract and then full text investigation. Medicine mistakes were most often reported in pediatric and neonatal patients. This was in consensus with literature information about the occurrences in other specialties. Fatal or life-threatening harm because of medicine mistakes was not often reported. However, most studies reported that the possible for the cases impairment as an outcome of a mistake is a significant problem. Investigation of types and level of medicine faults might results in steps towards the prevention of these faults and the improvement in the neonatal care quality and safety.

## Introduction

Medicine mistakes expressed as any avoidable action that might harm the patient as an outcome of professionals or patients and potentially more dangerous in the pediatric community rather in the mature population [**1**]. These faults might be associated with career action or to wellness care plans or policies, containing direction bums, medicine description, compounding, distributing, delivery, transportation, training, follow-up, and apply. Medicine mistakes sometimes lead to an adverse impact; many faults don’t result in damage or hurt, however show a low stage of security in wellbeing assistance [**2**]. Deliberation of different literature sources extended to whatever Medicine mistakes leads to valetudinarian complications and death in the pediatric community, as much of the study investigated the adult people [**3**]. Based on the National Coordinating Committee for Medicine mistakes recording and Restriction no trend rate for Medicine mistakes are tolerable, and for the Medicine mistakes prevention in wellness administration policies it should be to constantly improve [**4**]. Therefore, to reduce Medicine mistakes and progress in patient welfare by secure medication trainings, interventions are necessary. It seems a vital part of innocuous patient care, especially in the neonatal population avoiding Medicine mistakes. The medication stages manner involve obtaining/ ordering, reproducing/ checking, managing/ distributing and managing; Pediatric medicine mistakes could happen at any level of the system [**5**]. Nursing Interventions Class (NIC) define Medication administration as serving, delivering and assessing the efficacy of medicine and nonprescription medicines [**6**]. A prescription fault is any non-avoidable experience that might result in victim impairment whilst the medicine is in check of the wellness watch expert or cases [**4**]. An opposing medicine experience is an impairment that emerges as a result of a prescription or the absence of an expected medicine [**3**]. It should be remarked that not all medicine mistakes because of an opposing medication issue. As before discussed, Medicine mistakes happen more commonly in the pediatric harm and neonate community than in the mature population. Studies indicated that medicine mistakes in pediatrics were 3 times greater than in mature populations [**7**]. According to the outcomes of Antonow, out of two-hundred consecutive faults in a medical care clinic, 69.5% were pediatric patients [**5**]. A different range of parameters that could lead pediatric community more sensitive to medicine mistakes, and possible difficulties produced by medicine management are highlighted. Different dosage of the identical medicine are one of the main reasons for potential error. Several medicine for kids are produced in different liquid focuses, and several medicine contents might result in dosing faults [**8**]. Improper dosing is the usual known medicine mistake, which recorded in pediatrics [**9**]. One reason for the mentioned problem is limited regulated dosing for kids as related to adults. Besides, maximum pediatric medicine dosing is according to case mass, that needs a dosage consideration and could result in a fault. It is supposed to be the cause how kids are at a higher danger for opposing medicine issues. Kids diversify in mass, the volume of a body, and organ policy development; the whole of that influence their capability to metabolize and eliminate medicines [**9**]. Moreover, kids are usually inadequate to appropriately describe while they are encountering an opposing influence and have a restricted inside mental capability to shield medicine mistakes in association via adults [**8**]. Since previous research recommends that drug mistakes happen further periodically and are further troubling in the pediatric community, a regular survey of medication management failures and the pediatric community is guaranteed. The purpose of the regular survey is to explore for training-based studies, methodical studies and analysis reports on medicine mistakes in the pediatric inpatient community, involving the rate of experience, kind of management mistakes that happen and probable reasons of medicine mistakes in this age team. The scope of this research is the evaluation of nursing and medication research compared to medicine management mistakes in the pediatric and neonatal patients.

## Methods for Review

Electric Information; EMBASE, MEDLINE, Global Pharmaceutical Summaries and abbreviations, ASSIA, British Nursing Sign, CINAHL data bases are examined for studies printed among 1985 to December 2014. MeSH phrases applied originally for the orderly evaluation are “Medicine mistakes,” and the exploration is restricted to English-language books that are particular to the pediatric community. The reason backward restricting the research to treating journals is that nurses are usually the ones who manage medicines. Therefore it is considered that this would take all studies linked to medicine mistakes that happened over the management. The subjects and summaries examined for every paper, and manuscripts excluded if they did not associate with medicine security, containing medicine management or medicine mistakes, or if they did not concern to the medication management medication.

## Results 

Pharmaceutical mistakes have converted a significant concern amongst healthcare users in the previous decade. The 1999 medicine record Found “To Miss is Human: Creating a reliable wellness procedure” asserts that 45 up 98 thousand hospitalized American's fade per year of pharmaceutical mistakes [**10**]. This survival statement stirred expert, executive and cultural powers to work. For grown-ups, the recorded rate of faults in therapy via medicine varies up to 31 percent of full hospitalized recognition [**10**] or 5 percent of orders communicated. In pediatrics, though, this amount has been stated as great as 1 in 6.4 systems [**10**]. Further, there was a clearly improved ratio of medicine failure leading to hurt or loss in pediatric cases [**11**]. Medicine mistakes correlated via morbidity and fatality extended inpatient healthcare expense via an assessed $ 4650 each clinic access or around $2.8 million yearly for a 700-bedded education clinic [**12**]. The financial burden for all fields of healthcare of medication misadventures surpasses $100 billion yearly in the USA. The information about the incidence and financial effect of medicine mistakes is lacking in the developing world [**13**].

**Medicine mistakes division:**

Medicine mistakes according to many classifications according to different categories: 

A) According to level: planning (unambiguous medicine: imperfection of medicine sign; medicine content and mixture; program; Amount; dosage regimen; time; sign. Transcription (an equal proof of the prescript in the pharmaceutical report: mistake in medicine sign; medicine content and mixture; way; dosage; dosing regimen; medicine omission; unordered drug), dispensing medicine is concordant via designated remedy in nurse prescription plan (Unordered medicine (fault medicine); unordered dose; dose omission; wrong dose; wrong drug formulation) administering the appropriate medicine to the correct case in the proper direction and at the precise time, release reports, Eligible prescripts in medicinal studies are identical to orders in demobilization reports.

B) According to the origin: active which have an immediate effect like what happens if you give the patient adrenaline instead of furosemide for blood transfusion and he develops hypertension, tachycardia, latent error has a delayed effect and could be avoided since it happens. For instance, if the apothecary can not read the medicine as it is badly recorded, the prevention of the recurrence of this fault kind is to investigate later medicines more accurately and check the medicine. 

C) Based on severity: Potentially serious errors that can cause continuing infliction to cases and will improve hospitalization or the necessity for further processing like an overtreatment of potassium chloride in absolute parenteral nutrition; clinically significant errors can increase the requirement for patient monitoring e.g. Tazobactam 4 gm twice daily to a septic obese patient; clinically non-clear fault that does not hurt the case, Pantoprazole IV in a sick case who could eat.

D) Based on medicine mistake contents: Circumstances or issues that have the capability to make failure, a mistake happened except the medicine did not influence the case, a fault happened that transferred the case still didn't produce hurt to the case, a fault happened that lead to the requirement for improved case displaying, although no case hurt, a fault happened that led to the necessary for practice or interference and produced mortal hurt to the case, a fault happened that led to first or extended hospitalization and made acting harm to the patient, a mistake happened that lead to obvious hurt to the patient, a mistake happened that lead to near-death condition, and an fault happened that led to case fade [**14**,**15**].

**Predisposing factors for medicine mistakes**

Similar studies indicate that of the 25000 prescription fault records taken via the FDA, 12.5% of the faults linked to names [**11**]. Moreover, a current FDA research of 4 hundred mortality made via medicine mistakes realized that 5 percent of losses associated with established title disorder and 4 percent to general title disorder [**14**]. It is fair to extrapolate these data to prophesy that the rate of medicine mistakes in Canada related to data in the U.S., particularly as several supporters attempt for global flexibility in their outcomes branding. Faults might further happen because of incorrect choice of control methods [incorrect choice of insulin syringes]. In general, the Institute of Safe Medicine Practices (ISMP) identifies the following areas as potential causes of Medicine mistakes: failure in communication, medicine via alike titles, missing or mixed zero and decimal positions, employment of non-regular abstracts, bad medication delivery modes, complicated or badly produced technology, entrance to medicines via non-pharmacy group, job situation and place issues that result in an improved work pressure, miscalculations of dose, absence of case data, and absence of case realization of their treatment [**14**].

**Methods to detect Medicine mistakes**

Various approaches were utilized to identify the happening of medicine mistakes: Anonymous self-reports which were reported by the person himself or a witness, they have low cost but need realization; Incident reports - a legal report documented by the hospital staff, critical incident technique that involves an in-depth investigation of a large amount of personal mistakes to specify usual causal parameters, and close investigation of cases or questioning characters that have made the fault, disguised detection method while the witness completes the individual giving the medicine and witnesses the management of per dosage, dispensing error detection techniques detect the preparation before administration. The dispensing of medicine prescriptions at the drugstore can have multiple mistakes. The incorrect prescription can be provided, especially where medicines are labeled or packed likewise. Particular drugs are perceived to have queries since their titles are very alike. The drugstore can further give out the incorrect the drug dosage in some states. Utmost investigations of medicine faults just examined hospital medicine practice, and there is a huge quantity of medicines appointed in the doc’s positions and distributed via drugstores [**10**]. There are approximately 2.5 billion medicines administered via drugstores in 1998 in the US related to an approximated 3.75 billion medication management in clinics. Mistakes in Medicine and distributing are recognized though hard to quantify. For instance, the IOM statement cites an Australian research as 1988-1996, whatever determined that 2.4 to 3.6 % of clinic statements are because of medicine stories, of that 32 up 69 percent are avoidable. The prescriptions making most difficulties are cytotoxins, antihypertensives, cardiovascular pills, NSAIDs, and anticoagulants. [Huntley’s]. kids were not merely adults; they have different pharmacokinetics and pharmacodynamics, even among their population they vary according to their age groups which are classified as: preterm newborn infants < 37 weeks’ gestation, term newborn infants 0–27 days infants and toddlers 29 days to 24 months; kid 1–12 years, adolescents 13–18 or 19 years, vary according to the region [**3**]. Children have an unpredictable oral absorption, week painful muscular absorption, large outside space for covering reception rising toxicity hazard, moderately reduced renal elimination, smallest organ capability for prescription metabolism; all these lead to making drug handling most serious. Dosage adjustment based on the lifetime is a fast and reliable way for a remedy via a broad healing area similar artesunate and some antimicrobial to be presented. Nonetheless, not all kid have the best mass, then mass based consideration is further accurate, some drugs being adjusted by a different weight for each age groups, surface area based dose is preserved for serious drugs (cytotoxic drugs) taking into consideration the cardiac output, renal function, body fluid status, and child health. The most of pediatrics medications do not result in harm. Blum et al. [**16**] stated that just 0.2% of the errors could be classified as potentially lethal, whereas Folli [**17**] reported 6.5 percent as potentially lethal. Interestingly, no actual harm was reported to children in most of the epidemiological studies. This might be since the mistakes are identified and rectified since any hurt produced, but it can be because of the publication bias; some healthcare providers may be reluctant to publishing studies that report patients with serious harm. Cousins et al. [**18**] conducted an analysis of press reports highlighting the outcomes of 24 cases of pediatric Medicine mistakes. Most of the cases reported resulted in fatal consequences, hence making the news headlines.

**Types of error**

The usual kind of pediatric medicine mistakes is dosing errors, especially the tenfold error. The other pediatric Medicine mistakes that were stated in the previous research, include the following: wrong drug, wrong route of administration, mistake using, incorrect or out of time, incorrect management frequency, incorrect dose, incorrect case, medicine received by case with specified backward, medicines interfaces, intravenous conflict, ignoring mistakes, wrong rate of intravenous drug administration [**19**,**20**].

**Incidence Ratio of Medicine Mistakes**

Several variations realized via about the idea the studies received and described the occurrence ratio of medicine mistakes. Holdsworth et al., planned research to ascertain the extent and reasons of ADEs and possible ADEs in kids, and studied the results of those contests. The stated ADE repeating is 6% acceptance, and 7.5 in each 1000 case-days; the stated possible ADE rate was 8% acceptance, and 9.3 in each 1000 case-days. The ADEs that happened in the current research, 24 percent found to be dangerous or life-threatening [**3**]. Stratton et al., examined a supported pediatric representation and adult clinic nurses about their relationship thoughts of medicine mistakes listed on their parts [**21**]. The medicine mistakes ratio they attained for each 1000 case days are 14.8 on the pediatric part as related to 5.66 on the adult part. That was greater than the conclusions obtained by Holdsworth and might be justified by the diversity in their research plan involving their classification [**3**]. Ghaleb et al., [**10**] attended a systematic study that questioned the prevalence ratio of medicine mistakes and classified their outcomes based on if the trend ratio taken from chart analysis investigations, inevitable recording researches or measurement investigations. Among the 3 investigations involved that taken through chart study that was special to medicine management faults, the prevalence ratio are 0.15 percent doses applied are rambling and 23.5 percent management fault ratio [**10**]. The third research discovered that 3.9 percent of the 10 percent of cases related to faults are subjected to medicine management faults [**10**]. Variations in research plans and recording approach make it complex to describe and analyze the collected data by Ghaleb et al., that they realized to be correct and discussed in their research [**10**]. Amongst the 2 medicine management fault types of research involved via that are taken by automatic recording, the prevalence ratio is 14.7 events for each 100 receptions and 13.4 events for each 1000 case days [**10**]. 8 investigations that utilized research to identify medication management faults are further incorporated. The research examinations determined that stated medicine management fault ratio ranged from 0.6 and 27 percent of management. Another research examined nurses and determined that 40.3 percent of the partners showed they had witnessed a prescription fault in at concise one level of the manner over the past days [**5**]. Although it will be perfect to provide an accurate frequency ratio for medicine management faults in the pediatric inpatient community, that is hard because of variation in the recording. It was presented that some prevalence ratio is listed for each 100 selections, for each 1000 case days, and even as rates of entire management.

**Prescription Fault Reporting**

One research discovered that pediatric nurses determined that just 68% of medicine mistakes on their cases treatment parts listed [**21**]. The current research demanded causes as to why medicine mistakes were not reported, and concurrent personal and management, relevant causes are chosen via the partners, implying the necessity to acquire a part/ clinic situation mistake reporting supportive [**21**]. Another research examined examination issues via recorded event records and realized that the 89 medicine mistakes seen via the nurses, the participants showed that just 17 of the medicine mistakes led to the fulfillment of an event record [**5**]. Also, tt was discovered that the possibility of stopping a medicine mistake from stretching the case failed in the succeeding levels of the prescription procedure as before discussed, the possibility of a legally printed event record extended to the next levels of the prescription manner [**5**]. It was realized that out of the medicine mistakes that are not stopped and truly ended to the case, only 38 percent of the medicine mistakes that happened over the regulation / prescribing level recorded, 36 percent of the medicine mistakes that happened over the transcription/ confirmation stage recorded, 47 percent of the medicine mistakes that happened over the dispensing / distribution stage recorded, and 65 percent of the medicine mistakes that happened over the management stage recorded [**5**]. A research of Ferranti et al., examined a voluntary security recording process and an automated ADE monitoring policy about the repetition of ADEs ratio [**22**]. It was determined that the ADEs incidence was about a total ratio of 1.8 ADEs for each 1000 case days via the optional recording and 1.6 ADEs for each 1000 case days via the automated procedure. Although the ADEs rate is not analytically clear among the 2 methods, the scientist affirmed that the optional procedure presented better penetration to process breakdowns, like medicine prohibition, management mistakes, and failures in clinical displaying. These kinds of mistakes were not readily identified by automatic methods, indicating the necessity for a method that combines the robustness of both approaches therefore that the exposure of ADEs in the pediatric community could be maximized. A survey of the previous research recommended that consideration should be given to “near miss” medicine mistakes [**23**]. It is an issue that didn't cause hurt to a case nevertheless, has the possible to make abuse. Near misses have a great possibility of repeatedly occurring if they are not recorded, and if the reason for the near miss is not fixed. It is recommended that near miss medicine mistakes are recorded in the same condition as medicine mistakes [**23**]. A changed approach is required about listing and recording process for medicine mistakes [**24**]. Recording methods require being non-punitive hence that person sense comfortable recording and listing medicine mistakes. Moreover, an investigation of any recorded mistake and possible mistakes requires happening therefore that the underlying reason for the mistake in the entire system context can be notified [**24**]. To compile, this report determined that medicine mistakes lead to be under-recorded, usually because of punishment fear. It is also realized that optional recording granted exceptional perspicacity to policy missteps that result in the fault. Also, it is advised that near misses are recorded [**24**].

**Interruptions to limit medicine mistakes in kid**

During the last few years, professional organizations, government, and researchers have published many different guidelines and recommendations on prevention of Medicine mistakes. The following is a Summary of some important suggestions produced by the Pediatrics Committee American Academy on Medicine and Committee on Clinic Behaves, Found for Reliquary Medicine Training and the Pediatric Medicine Support Association [**25**-**27**]. Prescribers should be intimate via the pediatric patients and their medications, examining medicine sensitivities, communications, and contraindications and perceive these on the medicine plan, confirming that the case’s mass is true and writes the mass on any medication plan; write legible directions, not passing the suggested adult dose, calculation double checking by other staff is recommended, examining the medicine, dose, and case identification earlier treatment, particular amounts or dosages confirmation, information via case and caregiver. A sufficient amount of fitted organization and proper job conditions for secure and reliable and efficient application of drugs should be provided. Equipment [e.g. infusion pump] and measurement systems should be standardized to eliminate much of the hazards of evaluation mistakes as well as decrease the time needed for dose measurement. Barriers to medicine mistake recording should be removed; hospitals should develop and keep on a procedure to inform families of mistakes and send feedback data to employees. Children are more vulnerable to medicine mistakes than adults, but up to date, there have been a few studies in this field. One of the earliest studies is to define the currency of mistakes in the medicine method of a pediatric instruction clinic, and to contribute the way to attempting to error-proof the method as a long-term goal, but the sample size was too small to allow a further parceling; the methods utilized in the current research to examine the steps in the process required leaving a paper trail. The study was a prospective one done on a cohort, as mentioned by Kaushal et al., in 2001. The emergency rooms present a high risk for errors and need a quick and right decision. At the same time, this may need expert opinion. Keron et al., found this in 2002, and stated that in the ED of pediatric, trainees were more inclined to act ordering mistakes, and the complete severely sick cases were further expected to be constrained to ordering mistakes. They discovered that ordering mistakes recognized in 10 percent of the plans. The upcoming parameters are linked in unvaried investigations via an improved relationship of mistakes: cases observed among 4 and 8 AM (odds rate (OR): 2.45; 95 percent trust step (CI): 1.10 –5.50), cases via critical illness (OR: 2.53; 94 percent CI: 1.18 –5.41), medicine directed via a trainee [OR: 1.48; 94 percent CI: 1.03– 2.11], and cases observed over weekends [OR: 1.48; 94 percent CI: 1.04 –2.11]. There is a greater ratio of mistakes at the commencement of the educational year amongst neophytes [OR: 1.67; 94 percent CI: 1.06 –2.64). The logistic regression revealed a raised danger for mistakes while a medicine ordered via a neophyte [OR: 1.64; 94 percent CI: 1.06 –2.52 (also in severe sick cases [OR: 1.55; 94 percent CI: 1.06 –2.26] it was a retrospective cohort study, which might have preserved time, but it was only in summer, and this might have underestimated the errors because of the small proportion of hospital admission, adding to the fact that the thoughtful plan cannot identify several mistakes in medicine management. The other parts that can affect the medicine mistake ratio, like the communication among parents, cases, therapists, aides, sound, and simultaneous experiences in the ED, are not investigated since they are not recorded on the plan and can not be estimated properly. Hence, it just concentrated on ordering mistakes and reducing prescribing errors; clinics should prepare younger scholars concerning the origins of medicine dosing since they begin ordering, and support normal work in documentation [**28**]. The problem of prescribing drugs to a child is represented by the need of an accurate dosage adjustment, the younger the child, the most difficult the adjustment; that was evidenced by Chappell and Newman [**29**]. Another one was in the outpatient, finding that possible medicine dosing mistakes often happen in outpatient pediatrics. Investigations on the clinical affects of those possible mistakes and efficient failure stopping procedures were required. From 3 wellbeing maintenance systems, he found that about 16% of the kids are dispensed a medicine via a possible dosing mstake: 87% are possible overdoses, and 8% are possible underdoses. Amongst kid scaling <34 kg, just 66 percent of the dosage are given in the prescribed dosage intervals, and further than 1 percent are given at higher than double the approved greatest one. Analgesics are essentially expected to be possibly overdosed (16%), antiepileptic is extremely likely probably under dosed (20%). Possible mistake ratio are not lower at the site via an electric medicine order writer. However, prescribing mistakes can be avoidable; the general ordering difficulties reported during plan examination covering the fields of sensitivity documentation, hazardous discontinuing, and prescripts exchange, unclear reporting, and orders signing. Medication management was seen. The hazard fields specified involved failure to follow double-checking and case identity controlling process, weak management approach in the field of inhaled/ nebulised treatment, IV medicines and oral/ gastrostomy medicines and weak documentation. The research was undergone in summer, on one clinic via a small example area and the application of the undisguised observational method of medicine management. It might have possible impacts on the performance of the staff [**30**]. The physician may have a good plan and calculate his dose accurately, but nobody can read that, so using the computerized order, might help in preventing errors covered by Khowaj et al. [**31**]. 

## Conclusion

Medicine management mistakes are a main attention for the pediatric community. This paper proposed an investigation of medicine mistakes and secure medicine management training. Extra data about the pediatric community and particular parameters that cause this community responsive to medicine mistakes, were impersonated. Mistakes in dosage were discovered as a general cause to why medicine mistakes happen. There is a difference concerning medicine fault recording, as it was determined that medicine mistakes were not reported however the amount of them differed. Methods employed to list medicine mistakes differed too. It was discovered that the further the data recorded on the medicine mistake reported, the more it has a possible influence on resulting to a policy setting to stop before-mentioned mistakes from happening repeatedly. It was advised that recording methods are non-punitive, that nurses are not hesitant to list mistakes. Furthermore, more importance should be located on “near miss” medicine mistakes, since these happen regularly though are seldom recorded and might contribute a comprehensive penetration to policy defects. Lastly, interventions are determined to decrease medicine management mistakes and are corresponding to present suggestions for secure medicine management.
